# Supplementary material for: Rescue of pyrimidine-defective Pseudomonas aeruginosa through metabolic complementation
Source: Microbiol Spectr. 2024 Jul 11;12(8):e04226-23. doi: 10.1128/spectrum.04226-23 (PMC11302043; doi:10.1128/spectrum.04226-23)
Supplement: Fig. S1 — Rescue of pyrimidine-deficient mutants of P. aeruginosa through Uridine-5′-monophosphate. [file spectrum.04226-23-s0001.docx]

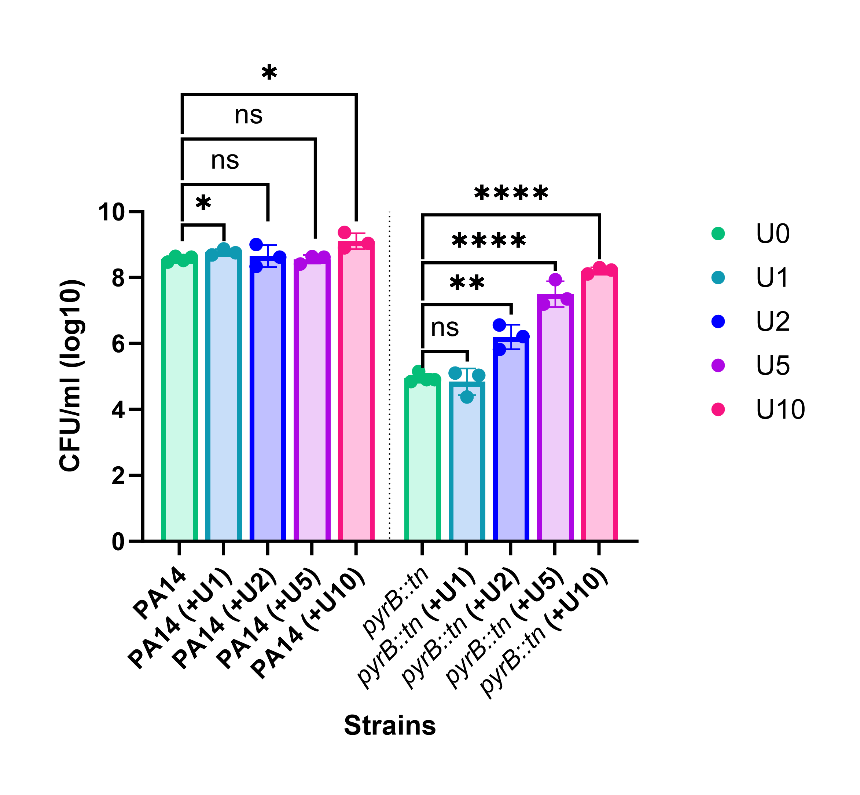


**Supplemental figure 1: Rescue of pyrimidine-deficient mutants of *P. aeruginosa* through Uridine-5′-monophosphate (UMP).** The lab reference strain, PA14, and the pyrimidine-deficient mutant *pyrB::tn* were exposed to various concentrations (0 – 10 mg/ml) of UMP. Error bars represent the SD of data obtained from three biological replicates conducted on different days. Each biological replicate consisted of three technical triplicates per day. ‘*’ designates *p* < 0.05, ‘**’ designates *p* < 0.005, ‘****’ designates *p* < 0.0001 as depicted by a two-tailed unpaired Student’s t-test, and ns denotes not significant (*p* > 0.05).
